# The Phosphodiesterase 10A Inhibitor PF-2545920 Enhances Hippocampal Excitability and Seizure Activity Involving the Upregulation of GluA1 and NR2A in Post-synaptic Densities

**DOI:** 10.3389/fnmol.2017.00100

**Published:** 2017-04-07

**Authors:** Yanke Zhang, Baobing Gao, Fangshuo Zheng, Shanshan Lu, Yun Li, Yan Xiong, Qin Yang, Yong Yang, Pengfei Fu, Fei Xiao, Xuefeng Wang

**Affiliations:** ^1^Department of Neurology, The First Affiliated Hospital of Chongqing Medical UniversityChongqing, China; ^2^Department of Neurology, Chongqing General HospitalChongqing, China; ^3^Center of Epilepsy, Beijing Institute for Brain DisordersBeijing, China; ^4^Chongqing Key Laboratory of NeurologyChongqing, China

**Keywords:** PF-2545920, status epilepticus, rat model, GluA1, NR2A

## Abstract

Phosphodiesterase regulates the homeostasis of cAMP and cGMP, which increase the strength of excitatory neural circuits and/or decrease inhibitory synaptic plasticity. Abnormally, synchronized synaptic transmission in the brain leads to seizures. A phosphodiesterase 10A (PDE10A) inhibitor PF-2545920 has recently attracted attention as a potential therapy for neurological and psychiatric disorders. We hypothesized that PF-2545920 plays an important role in status epilepticus (SE) and investigated the underlying mechanisms. PDE10A was primarily located in neurons, and PDE10A expression increased significantly in patients with temporal lobe epilepsy. PF-2545920 enhanced the hyperexcitability of pyramidal neurons in rat CA1, as measured by the frequency of action potentials and miniature excitatory post-synaptic current. GluA1 and NR2A expression also increased significantly in post-synaptic densities, with or without SE in rats treated with PF-2545920. The ratio of *p*-GluA1/GluA1 increased in the presence of PF-2545920 in groups with SE. Our results suggest that PF-2545920 facilitates seizure activity via the intracellular redistribution of GluA1 and NR2A in the hippocampus. The upregulation of *p*-GluA1 may play an important role in trafficking GluA1 to post-synaptic densities. The data suggest it would be detrimental to use the drug in seizure patients and might cause neuronal hyperexcitability in non-epileptic individuals.

## Introduction

Phosphodiesterases (PDEs), which hydrolyze cyclic AMP (cAMP) and/or cyclic guanosine monophosphate (cGMP), contain 11 isozymes encoded by 21 genes in mammals (Seeger et al., 2003; Bender and Beavo, 2006). Phosphodiesterase 10A (PDE10A) degrades both cAMP and cGMP but has a much higher affinity for cAMP (Soderling et al., 1999; Conti and Beavo, 2007; Francis et al., 2011). PDE10A is found in different multiple regions of the brain in mammalian species, including the striatal neuropil, substantia nigra, hippocampus, and cortex (Seeger et al., 2003). Several studies have demonstrated that PDE10A inhibitors upregulate cAMP and cGMP concentrations in the striatum (Siuciak et al., 2006; Grauer et al., 2009) and hippocampus (Liddie et al., 2012). PDE10A is found predominantly in the mammalian brain, suggesting that it has multiple functions in the central nervous system (CNS), especially in striatum-dependent behavioral phenomena (Siuciak et al., 2006; Leuti et al., 2013; Garcia et al., 2014). Recent studies demonstrated a putative role for PDE10A in the treatment of neurological and psychiatric disorders. Numerous PDE10A inhibitors have been verified as candidate drugs for the treatment of schizophrenia in animal or preclinical research (Siuciak et al., 2006; Giralt et al., 2013). PDE10A may be involved in other neurological and psychiatric disorders, including Huntington’s disease (Leuti et al., 2013; Ahmad et al., 2014; Niccolini et al., 2015), Parkinson’s disease (Garcia et al., 2014; Niccolini et al., 2015), addiction (Liddie et al., 2012; Logrip et al., 2014), and Lesch–Nyhan disease (Guibinga et al., 2013). PDE10A is also involved in improving spatial and recognition memories (Rodefer et al., 2012; Giralt et al., 2013) and appetite regulation (Piccart et al., 2011, 2013). Moreover, inhibitors of PDE10A may be useful for the treatment or prevention of colorectal cancer (Li et al., 2015). Cyclic nucleotides, including cAMP and cGMP, act as second messenger signaling molecules and participate in numerous cellular functions in the CNS, including neurotransmitter specification, axon guidance, and refinement of neuronal connectivity (Averaimo and Nicol, 2014; Mu et al., 2014). In addition, a previous study showed that activation of the cAMP–PKA pathway increases excitability and enhances epileptiform activity *in vitro* (Boulton et al., 1993; Chang et al., 2010; Lee, 2015).

Epilepsy is a human brain disorder characterized by recurrent seizures. Epilepsy is a devastating neurological disease with a worldwide prevalence of 1–2% (Hauser et al., 1993). Current therapies aim to control symptoms but are ineffective in the 20–30% of patients resistant to common antiepileptic drugs (AEDs) (French, 2007). Epilepsy is caused by many “insults,” such as trauma, stroke, inflammation, and status epilepticus (SE) (Duncan et al., 2006). These “insults” lead to progressive changes in brain structure and function and an imbalance of excitatory and inhibitory pathways (Curia et al., 2008). Aberrant excitatory synapses may result in neuronal hyperexcitability and recurrent seizures (Scharfman, 2007). SE, as a brain insult, can induce “epileptogenesis” (Loscher and Brandt, 2010). SE and hyperexcitability of neurons must be minimized. However, the molecular mechanisms underlying the pathogenesis of SE and epilepsy are not clear.

Papaverine, an intrinsic inhibitor of PDE10A, has been reported to cause seizures (Carhuapoma et al., 2001; Kahramaner et al., 2014), and the cAMP–PKA pathway plays a role in neuronal excitability of neurons (Boulton et al., 1993; Chang et al., 2010; Lee, 2015). Based on these data, we hypothesized that the PDE10A inhibitor PF-2545920 plays an important role in SE.

## Materials and Methods

### Subjects and Ethics

Adult male Sprague-Dawley (SD) rats from the Laboratory Animal Center of Chongqing Medical University weighing 200–230 g were used. Rats received water and food *ad libitum* and were maintained in a temperature-controlled, 12-h light/dark environment. Rats were randomly divided into experimental and control groups. All experiments were performed during the light phase, and the Chongqing Medical University Commission for Ethics of Experiments approved all procedures, which were conducted in accordance with international standards.

Twenty temporal neocortical samples from patients and ten control samples were randomly obtained from our human brain bank. Informed consent was obtained from patients and their lineal relatives. All protocols related to human subjects complied with the guidelines established by the Committee on Human Research at Chongqing Medical University and the National Institutes of Health. Detailed history, neurological examination, electroencephalogram (EEG) and neuroradiological studies were performed before surgery. Two or more neurologists diagnosed each patient, and the diagnosis of epilepsy was complied with the 1981 International Classification of Epileptic Seizures by the International League Against Epilepsy. All patients were refractory to maximal doses of three or more AEDs and required surgery. Normal temporal neocortex samples were obtained from patients diagnosed with brain trauma and no history of epilepsy or AEDs, who underwent neurosurgical intervention because of head trauma. **Tables 1**, **2** summarize the clinical features of temporal lobe epilepsy (TLE) patients and control subjects, respectively.

**Table 1:** Clinical characteristics of TLE patients.

Patients	Age (y)	Sex (M/F)	Course (y)	AEDs before surgery	Pathology	Resection tissue
1	28	M	12	CBZ, PHT, TPM	Gliosis	TNr
2	25	F	10	OXC, CBZ, VPA, PHT	Gliosis	TNl
3	33	F	15	LTG, TPM, PHT	NL	TNl
4	25	F	11	CBZ, LTG, TPM, PHT	Gliosis	TNr
5	20	M	13	VPA, CBZ, PB, PHT, LEV	NL	TNl
6	30	F	8	CBZ, VPA, PB, TPM	Gliosis	TNr
7	21	M	11	PB, CBZ, LEV, VPA, PHT	NL	TNl
8	23	M	10	PHT, CBZ, PB, VPA	Gliosis	TNl
9	26	M	12	CBZ, VPA, PB, TPM	NL	TNl
10	29	M	20	CBZ, VPA, TPM, LEV	NL	TNl
11	47	M	23	CBZ, VPA, PB, TPM, LTG	Gliosis	TNl
12	40	F	30	VPA, CBZ, PB, PHT	NL	TNl
13	19	M	7	OXC, VPA, CBZ, PHT	NL	TNl
14	28	F	12	PHT, PB, VPA, LEV	NL, Gliosis	TNr
15	26	M	8	VPA, CBZ, LEV, LTG	NL	TNr
16	29	F	5	PHT, TPM, LTG, VPA	NL	TNl
17	33	F	7	LEV, VPA, PHT	Gliosis	TNr
18	23	M	9	PB, VPA, TPM, LEV, LTG	NL	TNl
19	30	M	20	OXC, PB, CBZ, LEV	Gliosis	TNl
20	31	F	15	PB, VPA, PB, TPM, LEV	NL	TNr

*F, female; M, male; y, year; AEDs, antiepileptic drugs; VPA, valproic acid; PB, phenobarbital; CBZ, carbamazepine; PHT, phenytoin; GBP, gabapentin; LTG, lamotrigine; TPM, topamax; LEV, levetiracetam; OXC, oxcarbazepine; TN, temporal neocortex; l, left; r, right; NL, neuron loss.*

**Table 2:** Clinical characteristics of control patients.

Patients	Age (y)	Sex (M/F)	Etiology diagnosis	Resection tissue	Pathology
1	22	M	Trauma	TNl	N
2	30	M	Trauma	TNr	N
3	26	F	Trauma	TNl	N
4	21	F	Trauma	TNr	N
5	38	F	Trauma	TNl	N
6	31	M	Trauma	TNl	N
7	25	M	Trauma	TNr	N
8	22	M	Trauma	TNr	N
9	35	F	Trauma	TNl	N
10	43	M	Trauma	TNr	N

*F, female; M, male; y, year; TN, temporal neocortex; l, left; r, right; N, normal.*

### Drugs

2-((4-(1-Methyl-4-(pyridin-4-yl)-1H-pyrazol-3-yl) phenoxy) methyl) quinoline (PF-2545920, CAS NO: 1292799-56-4) was purchased from Selleck (Houston, TX, USA), dissolved in DMSO and diluted in 0.9% normal saline (NS). PF-2545920 or solvent control was administered intracerebroventricularly via a cannula (i.c.v., 0.02 μL/min, 10 μL) 30 min before behavior evaluation.

### Sample Preparation

Rats were anesthetized fully and decapitated 2 h after behavioral studies. Half of the rat or human brain tissues were placed in liquid nitrogen for Western blotting, and the remaining tissue was embedded in 4% paraformaldehyde for immunofluorescence.

### Surgical Process

The procedure used for intracerebroventricular cannulation and electrode implanting, with minor modifications, was described previously (Logrip et al., 2014; Xu et al., 2015) with minor modifications. Anesthetized rats were fixed in a stereotaxic apparatus (RWD Life Science, Co., Ltd, Shenzhen, China). The site of the left lateral ventricle was located using Paxinos and Watson: AP = 1.0 mm; ML = 1.5 mm on the left side; DV = 3.5 mm from the dura. A microwire array (a 2 × 8 array of platinum-iridium alloy wire, each wire 25 μm in diameter) was implanted into the right dorsal hippocampus (location: AP = 3.6 mm, ML = 2.8 mm, and DV = 3.6 mm). Two reference screws were implanted in the skull. All accessories were attached to the skull using cement. The following tests were performed 7 days after surgery and 3 days after environmental adaptation.

### Electrophysiology

Local field potentials (LFPs) were filtered (0.1–1000 Hz), 1000× amplified, and digitized at 4 kHz using an OmniPlex^®^ D Neural Data Acquisition System (Plexon, Dallas, TX, USA), as described previously (Gong et al., 2009; Xu et al., 2015).

Two- to three-week-old male SD rats were used for patch-clamp experiments. Coronal slices (380 μm thick) were cut using a vibratome (NVSLM1, Camden Instruments, Loughborough, UK). Slices were cut at 2°C in a solution containing the following: 26 mM NaHCO_3_, 22 mM sucrose, 11 mM glucose, 1 mM NaH_2_PO_4_, 3 mM KCl, 0.5 mM CaCl_2_, and 7 mM MgCl_2_ saturated with 95% O_2_ and 5% CO_2_. Slices recovered for 1 h at room temperature in artificial cerebral spinal fluid (ACSF) (pH 7.4): 1.25 mM NaH_2_PO_4_, 124 mM NaCl, 3 mM KCl, 26 mM NaHCO_3_, 2.5 mM CaCl_2_, 10 mM glucose, and 1.3 mM MgCl_2_, saturated with 95% O_2_ and 5% CO_2_ (Tang et al., 2015).

Pyramidal neurons in the hippocampal CA1 area were chosen for recording using inverted phase contrast microscopy (Nikon, Japan). Slices were stabilized for at least 5 min prior to recording after patch rupture. Epileptic discharges were characterized by activity potentials that manifested as continuous high-frequency spike discharges compared with activity potential in Mg^2+^-ACSF (Sombati and Delorenzo, 1995). To measure action potentials (APs), glass pipettes were filled with an internal solution: 17.5 mM KCl, 0.5 mM EGTA, 122.5 mM K-gluconate, 10 mM HEPES, and 4 mM ATP, pH adjusted to 7.2 with KOH. The bath solution was Mg^2+^-free ACSF with or without PF-2545920 (5 μM) during recording. To measure miniature excitatory post-synaptic currents (mEPSCs), glass microelectrodes were filled with the following solution: 17.5 mM CsCl, 10 mM HEPES, 4 mM ATP, 0.5 mM EGTA, 132.5 mM Cs-gluconate, and 5 mM QX-314. Bicuculline (10 μM) and tetrodotoxin (1 μM) were added to the Mg^2+^-free ACSF to record mEPSCs. For recording the miniature inhibitory post-synaptic currents (mIPSCs), glass microelectrodes were filled with the following solution recordings (pH 7.2, 275–290 mOsm): 100 mM CsCl, 1 mM MgCl_2_, 1 mM EGTA, 30 mM *N*-methyl-D-glucamine (NMG), 10 mM HEPES, 5 mM MgATP, 0.5 mM Na_2_GTP and 12 mM phosphocreatine. Slices were submerged and continuously perfused with Mg^2+^-free ACSF containing 6,7-dinitroquinoxaline-2,3(1H,4H)-dione (DNQX, 20 μM), D-2-amino-5-phosphonovalerate (APV, 40 μM) and tetrodotoxin (TTX, 1 μM) with or without PF-2545920 (5 μM). The membrane potential was maintained at -70 mV in voltage-clamp mode.

Signals were acquired using a Multi Clamp 700B amplifier (Axon, Sunnyvale, CA, USA) and recorded using pClamp 9.2 software (Molecular Devices, Sunnyvale, CA, USA). All recordings were filtered at 2 kHz and digitized at 10 kHz. Data were analyzed using Mini Analysis Software (Version 6.0.3; Synaptosoft, Decatur, GA, USA) and pClamp 9.2 software (Molecular Devices, Sunnyvale, CA, USA).

### Acute Rat Model of Seizures

Extrahippocampal lesions and hippocampus-restricted injuries in human TLE are comparable to pilocarpine-induced rat models, which are useful for investigating human TLE (Bonilha et al., 2010). Lithium chloride (127 mg/kg, i.p., Sigma, USA) and atropine sulfate (1 mg/kg, i.p.) were administered 20 h and 30 min prior to pilocarpine administration (30 mg/kg, i.p., Sigma), respectively. Diazepam (10 mg/kg, i.p.) was administered after 90 min of SE to terminate the convulsive seizures. Seizure activities were scored according to Racine’s standard criteria (Racine, 1972). Rats in the experimental group successfully kindled (levels 4 or more) and were considered the SE group. The control group was composed of rats that were not successfully kindled (levels < 4) and are the non-SE group. Electrographic seizure was defined as high frequency (>5 Hz), high amplitude (>2× baseline) and lasting more than 5 s (Palencia et al., 2012).

### Subcellular Fraction Preparation and Western Blot

Hippocampal tissue for subcellular fraction collection was collected 2 h after SE termination. The tissue was homogenized in ice-cold Tris-HCl buffer (30 mM, pH 7.4) containing 4 mM EDTA, 1 mM EGTA, 5 mM Na_4_P_2_O_7_, 1 mM Na_3_VO_4_, 100 μM (NH4)_6_MO_7_O_24_, 25 mM NaF, protease inhibitors (Roche Applied Science) and phosphatase inhibitors (Sigma-Aldrich). Homogenates were centrifuged at 700 *g* for 7 min at 4°C, and the supernatants were collected. Pellets were re-suspended and centrifuged at 4°C at 700 *g* for another 7 min. One portion of supernatants was collected as total homogenates, and other supernatants were centrifuged at 100,000 *g* for 1 h at 4°C. Pellets were re-suspended in the same buffer containing 0.5% Triton X-100 and incubated for 30 min at 4°C. The suspensions were layered on sucrose (1 M) and centrifuged at 100,000 × *g* for 1 h at 4°C. Triton-insoluble material (highly enriched in post-synaptic densities) sedimented through the sucrose layer and was re-suspended in the same buffer containing 1% SDS, then stored at -80°C (Dong et al., 2015; Zhang et al., 2016).

The same sample amounts (50 μg for total protein and 10 μg for subcellular fractions) were separated using 10% SDS-PAGE gels and transferred onto PVDF membranes. The membranes were blocked with 5% non-fat milk for 1 h at room temperature and blotted with the following primary antibodies: PDE10A (1:200, Santa Cruz, CA, USA), GluA1 (1:3000, Abcam), GluA2 (1:1000, Abcam), NR1 (1:3000, Abcam), NR2A (1:1000, Millipore), NR2B (1:1000, Millipore), PSD95 (1:500, Millipore), GABRA1 (1:500, Proteintech), *p*-GluA1 (Ser845, 1:1000, Abcam), CaMKII (1:1000, Abcam) and β-actin (1:5000, Abcam) overnight at 4°C. The blots were incubated with an HRP-conjugated goat anti-mouse or anti-rabbit antibody (1:3000) for 1 h at room temperature. The Enhanced Chemiluminescence Detection System (Amersham ECL) and Bio-Rad ChemiDoc XRS+ system were used for blot detection and analyses, respectively. Densitometry quantitation was performed using Quantity One Software (Bio-Rad Laboratories, Hercules, CA, USA). Total proteins were normalized to β-actin, and proteins from subcellular fractions were normalized to the same protein in total proteins.

### Immunofluorescence

Tissue sections (10 μm) were incubated in normal goat serum (Zhongshan Golden Bridge, Beijing, China) for 30 min followed by incubation with a mixture containing a PDE10A antibody (rabbit polyclonal antibody, 1:100), a microtubule-associated protein 2 (MAP2) antibody (chicken polyclonal antibody, 1:100, Zhongshan Golden Bridge) and a glial fibrillary acidic protein (GFAP) antibody (mouse polyclonal antibody, 1:100, Zhongshan Golden Bridge) overnight at 4°C. Sections were washed twice in PBS and incubated with a mixture of DyLight 488-conjugated goat anti-rabbit IgG (1:200, Zhongshan Golden Bridge), DyLight 549-conjugated goat anti-mouse IgG (1:200, Zhongshan Golden Bridge) and DyLight 405-conjugated goat anti-chicken IgG (1:200, Zhongshan Golden Bridge) in a darkroom for 90 min at 37°C. Tissue sections were mounted in 50% glycerol/PBS. Fluorescence was detected using laser scanning confocal microscopy (Leica Microsystems Heidelberg GmbH, Germany) on an Olympus IX 70 inverted microscope (Olympus, Japan) equipped with a Fluoview FVX confocal scan head. To determine the specificity of antibodies, PDE10A antibody (488 channel), GFAP antibody (549 channel) and microtubule-associated protein 2 (MAP2) antibody (405 channel) were replaced by PBS with the same protocols.

### Statistical Analysis

The data are presented as means ± SEM. Samples in each experiment were analyzed in triplicate. One-way ANOVA was used to compare data from more than two groups (SPSS 19.0). Paired-samples or independent-samples Student’s *t*-test were used for comparing two groups where appropriate. The χ^2^ test was used to compare gender differences between TLE patients and controls. *P* < 0.05 indicated statistical significance.

## Results

### Clinical Characteristics of Human Subjects

The 20 patients (9 females, 11 males) in the TLE group had a mean age of 28.3 ± 1.489 years and an average disease course of 12.9 ± 1.382 years. The 10 cases (four females and six males) in the control group had a mean age of 29.3 ± 2.367 years. There were no significant differences in age or sex between the TLE group and control group (*P* > 0.05).

### PDE10A Expression in the Hippocampus and Cortex of the Rat Model

**FIGURE 1:**
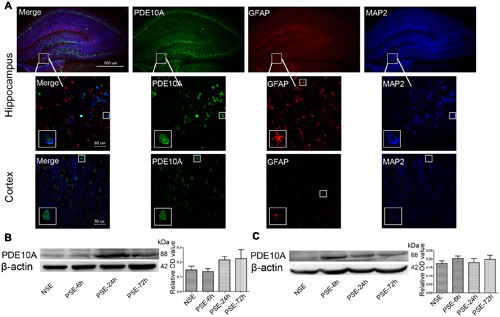
**PDE10A expression in the hippocampus and cortex of rats. (A)** Triple-label immunofluorescence demonstrated that PDE10A (green) and GFAP (red) were not co-expressed in astrocytes, but PDE10A (green) and MAP2 (blue) were co-expressed in neurons (*n* = 5). The white squares indicate positive cells in the cortex or hippocampus of rats. Representative Western blot images of hippocampus **(B)** and cortex **(C)** of rats at different time points post-status epilepticus. PDE10A increased after SE with a trend, but not significantly (non-SE; 6 h post-SE, PSE-6; 24 h post-SE, PSE-24; 72 h post-SE, PSE-72; *n* = 5, *P* > 0.05).

Triple-labeled immunofluorescence staining revealed that PDE10A (green) and MAP2 (blue) were co-expressed in neurons in the hippocampus and cortex of rat models but not with GFAP (red) in astrocytes (**Figure 1A**). We also examined the levels of PDE10A protein in the hippocampus and cortex of rat models at different time points after SE using Western blotting to confirm PDE10A expression in our animal model. PDE10A expression increased in the hippocampus (**Figure 1B**, NSE: 0.148 ± 0.025, PSE-6 h: 0.137 ± 0.019, PSE-24 h: 0.217 ± 0.022, PSE-72 h: 0.224 ± 0.061; *n* = 5, *P* > 0.05) and cortex (**Figure 1C**, NSE: 0.174 ± 0.015, PSE-6 h: 0.203 ± 0.015, PSE-24 h: 0.18 ± 0.023, PSE-72 h: 0.198 ± 0.025; *n* = 5, *P* > 0.05), but not significantly. The control group was composed of rats that were not successfully kindled (levels < 4) and recorded as the non-SE group.

### PDE10A Expression in the Neocortex of TLE Patients

**FIGURE 2:**
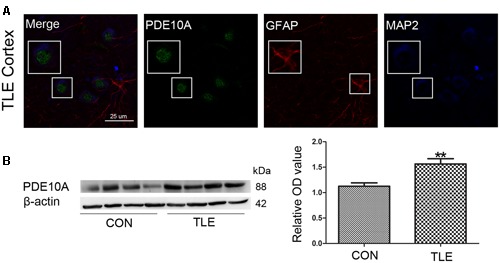
**PDE10A expression in the temporal cortex of TLE patients. (A)** Triple-label immunofluorescence demonstrated that PDE10A (green) and GFAP (red) were not co-expressed in astrocytes, but PDE10A (green) and MAP2 (blue) were co-expressed in the neurons of patient cortex (*n* = 10). The white squares indicate positive cells. **(B)** Representative Western blots revealed PDE10A expression in TLE patients and controls. The mean OD ratio of PDE10A relative to β-actin was significantly higher in the TLE group compared with the control group (**P < 0.01).

Data from our animal studies suggested that PDE10A is involved in the acute seizure model; therefore, we hypothesized that it may play a role in epilepsy patients. We examined PDE10A expression using Western blotting and immunofluorescence. Immunofluorescence staining demonstrated that PDE10A (green) and MAP2 (blue) are co-expressed in neurons in the neocortex of TLE patients but not with GFAP (red) in astrocytes (**Figure 2A**). Negative control for all channels was shown in the (**Supplementary Material Figure S1**). We performed Western blotting to examine alternations in PDE10A expression in human sections. PDE10A expression increased significantly in TLE patients compared with control groups (*P* < 0.05) (**Figure 2B**).

### Effect of PF-2545920 on Epileptiform Discharge and Epileptic Seizure

**FIGURE 3:**
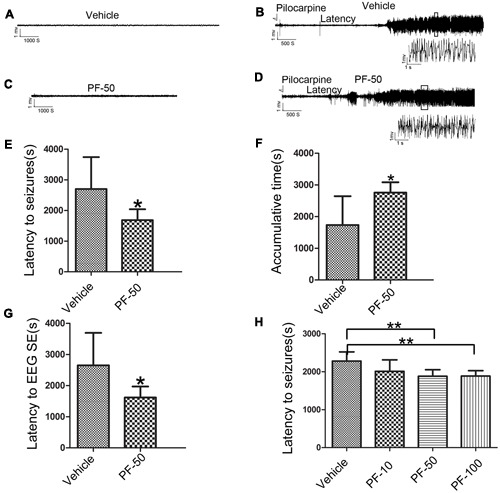
**Effect of PF-2545920 on epileptic seizure. (A)** Representative figure of LFP under the treatment of vehicle alone. **(B)** Representative recordings of Licl-Pilocarpine-induced status epilepticus in the vehicle group. **(C)** Representative figure of LFP under PF-2545920 treatment (50 μM, 10 μL, i.c.v., PF-50). **(D)** Representative recordings of Licl-Pilocarpine-induced status epilepticus after PF-2545920 administration (50 μM, 10 μL, i.c.v., PF-50). **(E)** Behavioral evaluation revealed that the latency time in the PF-2545920-treated group was significantly decreased compared with the vehicle-treated group (50 μM, 10 μL, i.c.v., PF-50; *n* = 8–9, *P < 0.05). **(F)** Behavioral evaluation demonstrated that the cumulative time of generalized tonic clonic seizures in PF-2545920-treated group increased significantly compared with the vehicle-treated group (50 μM, 10 μL, i.c.v., PF-50; *n* = 8–9, *P < 0.05). **(G)** EEG evaluation of latency time to full electrographic SE in PF-2545920-treated group was statistically decreased compared with the vehicle-treated group (50 μM, 10 μL, i.c.v., PF-50; *n* = 8–9, *P < 0.05). **(H)** Behavioral evaluation demonstrated that latency time in PF-2545920-treated groups at 50 and 100 μM were statistically decreased compared with the vehicle-treated group (10 μM, 10 μL, PF-10; 50 μM, 10 μL, PF-50; 100 μM, 10 μL, PF-100; *n* = 7, **P < 0.01).

We performed behavioral and LFP studies to examine whether the administration of PF-2545920 alone induced spontaneous seizures and epileptiform discharge. Vehicle or PF-2545920 (50 μM) was administrated and animals were monitored for 6 h (10 μL, i.c.v., *n* = 5). Vehicle or PF-2545920 alone did not induce spontaneous seizures or epileptic discharges or any other abnormalities (**Figures 3A,C**). We administered PF-2545920 once (50 μM, 10 μL, intracerebroventricularly) (PF-50) prior to pilocarpine application to examine the role of PF-2545920 in the progression of epileptic seizure. The latency time was significantly shortened in the PF-50 group compared with the vehicle group (**Figure 3E**, 1683.33 ± 118.91 s, *n* = 9 vs. 2703.13 ± 367.28 s, *n* = 8; *P* < 0.05). The cumulative time was significantly increased in the PF-50 group compared with the vehicle group (**Figure 3F**, 2757 ± 109.86 s, *n* = 9 vs. 1734.38 ± 321.91 s, *n* = 8; *P* < 0.05).

Status epilepticus is characterized by convulsions and the synchronized discharges of a population of neurons. Therefore, we used *in vivo* LFP recording to confirm the effect of PF-50 on LFP. PF-50 treatment dramatically shortened the latency to full electrographic SE (**Figure 3G**, 1619.22 ± 117.82 s, *n* = 9 vs. 2650.13 ± 369.68s, *n* = 8; *P* < 0.05). **Figures 3B,D** show the typical figures of LFP after pilocarpine with (**Figure 3D**) or without (**Figure 3B**) PF-50. Using the same protocol, we investigated the effect of different concentrations of PF-2545920 on SE. There was no significant difference in latency time between the vehicle group and PF group at 10 μM. However, there was a significant difference between groups treated with PF-50 μM or PF-100 μM compared with the vehicle group (**Figure 3H**, *P* < 0.01). These results indicate that PF-2545920 exhibits proconvulsant effects in a dose-dependent manner with a ceiling effect.

### PF-2545920 Induced Neuronal Hyperexcitability

**FIGURE 4:**
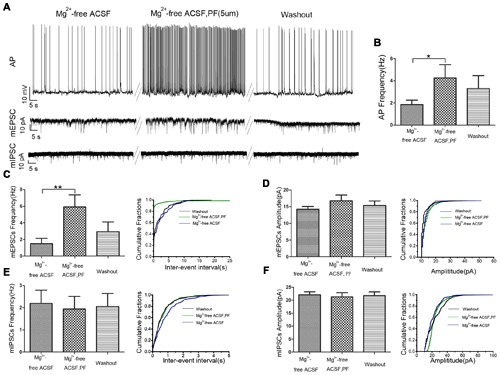
**PF-2545920 increases the excitability of pyramidal neurons in the CA1 region. (A)** Representative traces of action potentials (APs), mEPSCs, mIPSCs before and after the perfusion of PF-2545920. **(B)** Frequency of APs increased significantly in pyramidal neurons after perfusion of PF-2545920 (data are means ± SEM, *n* = 10, *P < 0.05). **(C)** Frequency of mEPSC increased significantly after PF-2545920 perfusion (data are means ± SEM, *n* = 10, **P < 0.01), and its representative cumulative fractions. **(D)** Amplitude of mEPSC was not altered after PF-2545920 treatment (data are means ± SEM, *n* = 10, *P* > 0.05), and its representative cumulative fractions. **(E)** Data demonstrated no significant difference in the frequency of mIPSCs after PF-2545920 treatment (data are means ± SEM, *n* = 10, *P* > 0.05), and its representative cumulative fractions. **(F)** Data revealed no significant difference in the amplitude of mIPSC after PF-2545920 treatment (data are means ± SEM, *n* = 10, *P* > 0.05), and its representative cumulative fractions.

We used whole cell patch-clamp electrophysiology on CA1 pyramidal neurons in rat hippocampal slices perfused with Mg^2+^-free ACSF to further evaluate the role of PF-2545920 in the hippocampus and verify observations from the behavioral tests. **Figure 4A** show the typical features of APs, mEPSCs, and mIPSCs, respectively, in pyramidal neurons before or after perfusion with PF-2545920 (5 μM). The frequency of APs from pyramidal neurons after PF-2545920 (5 μM) perfusion increased significantly compared with the frequency immediately prior to perfusion with Mg^2+^-free ACSF (**Figure 4B**, 4.26 ± 1.19 Hz vs. 1.86 ± 0.39 Hz; *n* = 10, *P* < 0.05). To determine whether the imbalance of excitatory and inhibitory transmission may cause neuronal hyperexcitability, we recorded mEPSCs and mIPSCs. Brain slices perfused with PF-2545920 demonstrated a significant increase in the frequency of mEPSCs (**Figure 4C**, 5.92 ± 1.45 Hz vs. 1.50 ± 0.64 Hz; *n* = 10, *P* < 0.05). No significant differences between groups were found in the amplitude of mEPSCs (**Figure 4D**) or the frequency (**Figure 4E**) or amplitude of mIPSCs (**Figure 4F**). The corresponding cumulative fraction of mEPSCs or mIPSCs confirmed our results. There was no significant difference in the frequency of AP or the frequency or amplitude of mEPSCs and mIPSCs prior to perfusion or after washout (**Figure 4A**, *n* = 10, *P* > 0.05). The electrophysiological data were consistent with and confirmed the findings of our animal behavior study.

### GluA1 and NR2A Levels Increased in PSD

**FIGURE 5:**
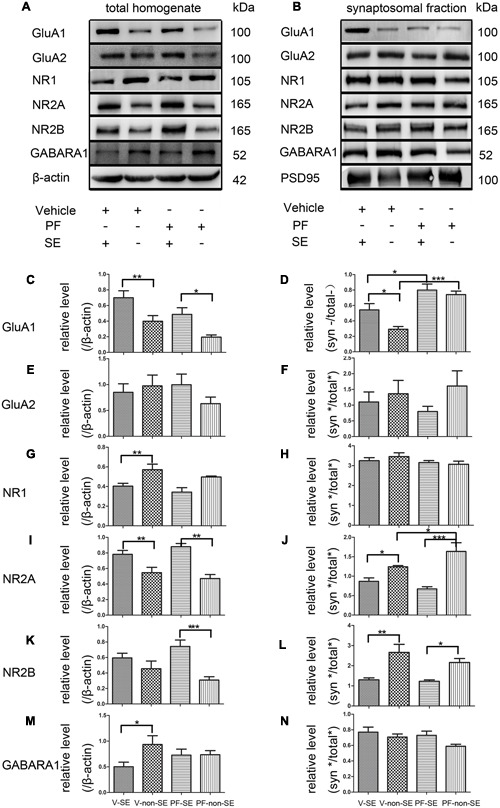
**Effect of PF-2545920 on AMPAR, NMDAR, and GABAR expression in total homogenates and post-synaptic densities.** Western blot analyses of hippocampus extracts from rats across groups: vehicle-treated rats with SE group (V-SE), vehicle-treated rats without SE group (V-non-SE), PF-2545920 (50 μM, 10 μL) (PF-50)-treated rats with SE group (PF-SE), and PF-50 treated rats without SE group (PF-non-SE). **(A)** Representative Western blots of total tissue homogenates. **(B)** Representative Western blots of subcellular fractions. **(C)** SE significantly increased GluA1 expression in total homogenates with or without SE. There was no significant difference in GluA1 expression between groups with or without PF-2545920 treatment. **(D)** The ratio of synaptic/total GluA1 increased significantly in the PF-2545920-treated group compared with the vehicle-treated group with or without SE. **(E)** There was no significant difference in GluA2 expression between groups treated with or without PF or the synaptic/total ratio **(F)**. **(G)** Seizures significantly decreased NR1 expression in total homogenates. There was no significant difference between groups treated with or without PF. **(H)** There was no significant difference in NR1 expression between groups. **(I)** Seizures significantly increased NR2A expression in total homogenates. However, there was no significant difference in NR2A expression between groups treated with PF compared with groups without PF treatment. **(J)** Seizures significantly decreased the synaptic/total ratio of NR2A. PF significantly increased the synaptic/total ratio of NR2A between groups without SE. **(K)** Seizures significantly increased NR2B expression in total homogenates in groups without SE. **(L)** Seizures significantly decreased the synaptic/total ratio of NR2B in groups with or without SE. **(M)** Seizures significantly decreased GABARA1 expression in groups with SE. **(N)** There was no significant difference in the synaptic/total ratio of GABARA1 between groups (*n* = 5, *P < 0.05; **P < 0.01; ***P < 0.001).

Total and synaptic NMDAR, AMPAR, and GABAR subunit compositions are not static but change dynamically in response to a variety of neuronal activity, including SE (Rajasekaran et al., 2013; Loddenkemper et al., 2014). Activity dynamically regulates the functional multiprotein units of the PSD via local protein turnover (Ehlers, 2003). Imbalance between inhibition and excitation is one of the main causes of seizures (Scharfman, 2007). We investigated the total (**Figure 5A**) and synaptic (**Figure 5B**) expression levels of the main subunits of AMPAR (GluA1 and GluA2), NMDAR (NR1, NR2A, and NR2B) and GABARA1 to delineate the molecular mechanisms linking the inhibition of PDE10A to the imbalance of excitatory and inhibitory transmission. We compared subunit expression between vehicle-treated rats with SE (V-SE group), vehicle-treated rats without SE (V-non-SE group), PF-50-treated rats with SE (PF-SE group), and PF-50-treated rats without SE (PF-non-SE group) in total lysates and their synaptic/total ratio (*n* = 5 in each group, one-way ANOVA followed by LSD tests). In total protein homogenates, the data showed that PF-2545920 did not affect the levels of GluA1 (V-SE group: 0.7 ± 0.089 vs. PF-SE group: 0.486 ± 0.086, *P* > 0.05; V-non-SE group: 0.4 ± 0.072 vs. PF-non-SE group: 0.196 ± 0.028, *P* > 0.05, **Figure 5C**). However, seizures increased the expression of GluA1 (V-SE: 0.7 ± 0.089 vs. V-non-SE: 0.4 ± 0.072; PF-SE: 0.486 ± 0.086 vs. PF-non-SE: 0.196 ± 0.028, *P* < 0.05). Unexpectedly, PF-2540920 increased the synaptic/total ratio significantly (V-SE: 0.543 ± 0.08 vs. PF-SE: 0.799 ± 0.079; V-non-SE: 0.292 ± 0.037 vs.PF-non-SE: 0.742 ± 0.045, *P* < 0.05, **Figure 5D**). These results indicate that PF-2545920 induced the redistribution of GluA1 to post-synaptic densities. Notably, GluA2 expression was not altered in total lysates (**Figure 5E**) or the synaptic/total ratio (**Figure 5F**, *P* > 0.05). The levels of other proteins involved in synaptic function, including NMDA receptor subunits NR1, NR2A, and NR2B, were not altered after PF-2545920 treatment in total lysates (**Figures 5G,I,K**). No significant differences in the synaptic/total ratio of NR1 and NR2B were found related to the effect of PF-2545920 (**Figures 5H,L**). However, NR2A was significantly increased in the synaptic/total ratio after PF-2545920 treatment in groups without SE (**Figure 5J**). The results of the total lysis suggest that PF-2545920 affected the distribution of NR2A in the PSD. The primary subunit of GABARs, GABARA1, was also investigated in homogenates and subcellular fractions. Although the overall levels of GABARA1 were reduced after SE, PF-2545920 did not alter the distribution of GABARA1 (**Figures 5M,N**), which was consistent with our findings using whole cell patch-clamp. These data demonstrated significant alterations in GluA1 and NR2A in the subcellular fractions, further supporting our electrophysiological data and correlating, in part, with our behavioral data.

### *p*-GluA1 and CaMKII Expression in Rat Hippocampus

**FIGURE 6:**
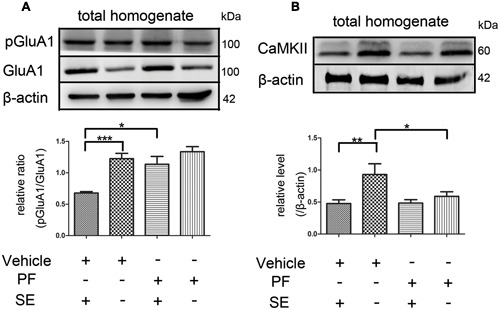
**The *p*-GluA1Ser845/GluA1 ratio and CaMKII in rat hippocampus. (A)** Representative Western blot shows that seizures significantly decreased the ratios of *p*-GluA1Ser845 relative to GluA1 in total homogenates. PF significantly increased the ratios of *p*-GluA1Ser845 relative to GluA1 in groups with SE (*n* = 5, *P < 0.05; ***P < 0.001). **(B)** Representative Western blot shows that seizures significantly decreased the expression of CaMKII in total homogenates. PF significantly decreased the expression of CaMKII in groups without SE (*n* = 5, *P < 0.05; **P < 0.01).

PF-2545920 affects the phosphorylation of GluA1 at Ser845 (*p*-GluA1Ser845) (Grauer et al., 2009). Our study demonstrated that the ratio of *p*-GluA1Ser845/GluA1 in PF-SE was significantly higher than the V-SE group (**Figure 6A**, 1.135 ± 0.126 vs. 0.679 ± 0.022; *n* = 5, *P* < 0.05). Previous studies have demonstrated that CaMKII participates in the synaptic modification of synaptic receptors (NMDARs, AMPARs). Our data revealed that PF-2545920 downregulated CaMKII expression compared with the non-SE groups (**Figure 6B**, 0.588 ± 0.073 vs. 0.93 ± 0.167; *n* = 5, *P* < 0.05).

## Discussion

We present the following results in this work: (i) PF-2545920 exerted proconvulsant effects; (ii) PF-2545920 induced hyperexcitability of pyramidal neurons in CA1; (iii) PF-2545920 redistributed GluA1 and NR2A to the PSD; (iv) PDE10A expression increased significantly in TLE patients; and (v) PF-2545920 upregulated the *p*-GluA1Ser845/GluA1 ratio.

Our data suggest that PDE10A expression was upregulated in the cortex of patients with epilepsy. The exact role of upregulated PDE10A in TLE patients is unknown. We found that PDE10A was primarily located in neurons in our experimental rat model and in human patients, which is consistent with a previous study (Seeger et al., 2003). However, the hippocampus is the most widely studied brain region in human and experimental epilepsy (Thom, 2014). Electrophysiology experiments were performed to examine the effect of PF-2545920 on neuronal hyperexcitability, revealing that PF-2545920 increased excitatory, but not inhibitory, synaptic transmission. This imbalance of excitatory and inhibitory conductance may lead to the proconvulsant effects of PF-2545920, consistent with a previous study (Scharfman, 2007).

Synapse formation is essential for the physiology and pathology of neuronal circuitry in the brain, and its impairment is a primary cause of epilepsy (Sutula et al., 1988; Rakhade and Jensen, 2009). The PSD, which contains receptors and signaling proteins, is the primary structure underlying synaptic plasticity (Scannevin and Huganir, 2000). Accumulating evidence reveals that synaptic NMDAR, AMPAR, and GABAR are not static but change dynamically in response to neuronal activity. These changes contribute to neuropsychiatric disorders when the balance is disturbed under pathological conditions (Lau and Zukin, 2007). Russwurm et al. (2015) found that in the striatum, PDE10A acts as a post-synaptic signaling element and is co-immunoprecipitated with PSD95 and NMDA receptor subunits. Notably, biochemical data showed that GluA1 and NR2A were trafficked to the PSD after PF-2545920 treatment and electrophysiological data showed an increase of mEPSCs. We combined biochemical and electrophysiological research methods because the biochemical results allowed us to identify specific changes in the receptor while the electrophysiological data provided the direct evidence of neuronal hyperexcitability. There was no significant difference in GluA2 levels among the treatment and control groups. This result is somewhat surprising because it has been suggested that increased levels of synaptic GluA2 homomeric AMPAR are important for SE (Rajasekaran et al., 2012). The exact mechanisms are not clear, but a growing body of evidence has convincingly demonstrated that AMPARs lacking GluA2 exhibit distinct biophysical characteristics, including greater conductance, calcium permeability, and inwardly rectifying currents (Zaccara et al., 2013). A prior study demonstrated that the selective activation of NR2A-containing NMDARs increases BDNF gene expression, which contributes to the development of epilepsy (Chen et al., 2007). However, SE in rats or patients affects the surface expression of GluA1 and NR2A or other subunits (Rajasekaran et al., 2013; Loddenkemper et al., 2014). Therefore, the aberrant trafficking of GluA1- and NR2A-containing receptors may be attributable to SE or/and PF-2545920. Data from our biochemical and behavioral experiments suggest that the proconvulsant effects are attributable to the redistribution of GluA1- and NR2A-containing receptors mediated by PF-2545920.

GluA1 contains phosphorylation sites for CaMKII, PKC and PKA, which regulate its synaptic insertion (Anggono and Huganir, 2012). We examined *p*-GluA1 expression to uncover the mechanism regulating redistribution of GluA1. Our experiments demonstrated a significant increase in *p*-GluA1Ser845 after PF-2545920 treatment. This result is consistent with a previous study demonstrating that a PDE10A inhibitor regulated the phosphorylation state of presynaptic- or post-synaptic-associated complexes (Nishi et al., 2008). Notably, GluA1 phosphorylation may participate in neurotransmitter transmission and strengthening of seizure activity via the potentiation of currents induced through AMPA receptors (Benke et al., 1998; Derkach et al., 1999). CaMKII is a post-synaptic protein involved in synaptic modification, and it is critically required for the synaptic recruitment of AMPARs and NMDARs (Opazo et al., 2010). In addition, Opazo et al. (2010) showed that exocytosis and/or trapping of AMPAR-containing vesicles to the PSD in activated synapses may be regulated in a CaMKII-dependent manner. The expression of CaMKII was downregulated, which suggests that CaMKII does not play an important role in the phosphorylation of the GluA1 subunit after PF-2545920 treatment. However, the downregulation of CaMKII might prevent GluA1 redistribution to the PSD. These findings suggest that *p*-GluA1Ser845 participates in the redistribution of GluA1 via the cAMP/PKA signaling pathway. Russwurm et al. (2015) found that in the striatum, PDE10A is co-immunoprecipitated with PSD95 and NMDA receptor subunits. Further studies are necessary to reveal the exact trafficking mechanism of NR2A in our study. However, NMDARs in the PSD differ in their subunit compositions and are differentially regulated in response to changes in phosphorylation (Barria and Malinow, 2002); further studies is required. Previous study showed that PF-2545920 upregulates cAMP concentration (Siuciak et al., 2006; Grauer et al., 2009; Liddie et al., 2012), while cAMP modulate epileptiform after discharge generation in rat hippocampal slices (Higashima et al., 2002). Our data showed PF-2545920 enhances the excitability of pyramidal neurons and seizure activity possibly via activation of the cAMP–PKA pathway.

Status epilepticus is a life-threatening form of seizure activity that represents a major medical emergency associated with significant morbidity and mortality (Chen and Wasterlain, 2006). Preclinical studies have recently investigated PF-2545920 as candidate drug. Volunteers participating in early clinical trials rarely experience serious injuries, but these events are very severe when they do occur (Bass et al., 2004). Therefore, it is essential to identify any proconvulsant activity for drugs being studied (Porsolt et al., 2002). Additional studies should be performed to characterize the physiological, pharmacological, and behavioral actions of PF-2545920 in humans and other species exposed to this drug. The upregulation of PDE10A in TLE patients and the trend of upregulation in rats after SE compared with controls (trauma patients or rats that were not successfully kindled) might modulate or decrease seizures. There are some limitations in our study. While no significant alterations to GluA2-containing receptors were detected, we cannot exclude the involvement of GluA2-containing AMPARs in PF-2545920-mediated proconvulsant functions or NR1 and NR2B. Ethical limitations preclude the absolute acquisition of absolutely normal brain tissues and human hippocampus for investigation. Recently, Harada et al. (2017) found an inhibitor targeting PDE10A, which suppressed seizure frequency. Although, the precise reasons for this discrepancy remain unclear, there are some possibilities: (i) The animal model used in their work, “R6/2 mouse model of Huntington’s disease,” has a background of neurodegeneration and spontaneous seizures under stress. In contrast our animal model is a healthy rat injected with pilocarpine to initiate SE. (ii) Their study, which shows that repeated treatment with TAK-063 prevents striatal neurodegeneration, is focused on the mechanism by which TAK-063 blocks the reduction of BDNF levels. Our study showed that PF-2545920 enhances the excitability of pyramidal neurons and seizure activity possibly via activation of the cAMP–PKA pathway (Boulton et al., 1993; Chang et al., 2010; Lee, 2015). (iii) TAK-063, which has a faster off-rate, induces similar levels of activation in medium spiny neurons (MSNs) in the indirect pathway, while MSNs in the direct pathway are only partially activated (Suzuki et al., 2016). The role of TAK-603 in the hippocampus may differ from PF-2545920. If so, TAK-063 is a better choice for neurological disease and should be studied further.

## Conclusion

Our study revealed that PF-2545920 has a robust proconvulsant effect and induced neuronal hyperexcitability, which is accompanied by changes in synaptic composition. We also demonstrated that PF-2545920 enhances the phosphorylation of GluA1 at Ser845. To our knowledge, this is the first study to investigate the effects of PF-2545920 on SE. A full understanding of this drug will guide its clinical use.

## Author Contributions

BG and XW: conceived and designed the experiments. YZ, YX, YL, PF, and QY: performed the experiments and the statistical analysis. FZ, SL: analyzed and collected the data. FX and YY: wrote the manuscript. All authors contributed to preparation of the manuscript and approved the final contributions.

## Conflict of Interest Statement

The authors declare that the research was conducted in the absence of any commercial or financial relationships that could be construed as a potential conflict of interest.

## Supplementary Material

FIGURE S1 **Negative control for each channel**. PDE10A antibody (488 channel), glial fibrillary acidic protein (GFAP) antibody (549 channel) and microtubule-associated protein 2 (MAP2) antibody (405 channel) were replaced by phosphate buffered saline (PBS). In the staining, no neurons or glia was marked separately.
