# Supplementary figures and images for: The Phosphodiesterase 10A Inhibitor PF-2545920 Enhances Hippocampal Excitability and Seizure Activity Involving the Upregulation of GluA1 and NR2A in Post-synaptic Densities
Source: Front Mol Neurosci. 2017 Apr 7;10:100. doi: 10.3389/fnmol.2017.00100 (PMC5383654; doi:10.3389/fnmol.2017.00100)

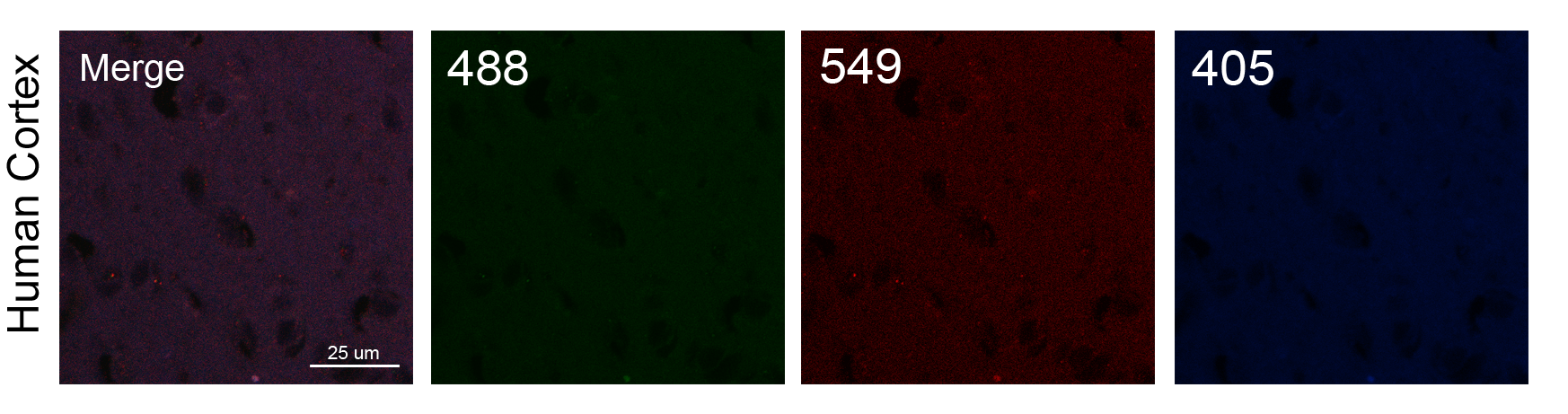

Supplement: FIGURE S1 — Negative control for each channel. PDE10A antibody (488 channel), glial fibrillary acidic protein (GFAP) antibody (549 channel) and microtubule-associated protein 2 (MAP2) antibody (405 channel) were replaced by phosphate buffered saline (PBS). In the staining, no neurons or glia was marked separately. [file Image_1.tif]
